# Rhenium‐Based Complexes and in Vivo Testing: A Brief History

**DOI:** 10.1002/cbic.202000117

**Published:** 2020-04-14

**Authors:** Miles S. Capper, Hollie Packman, Mark Rehkämper

**Affiliations:** ^1^ Department of Earth Science and Engineering Imperial College London, Royal School of Mines Prince Consort Rd Kensington London SW7 2AZ UK

**Keywords:** Antitumor, chemotherapy, cisplatin, in vivo, rhenium

## Abstract

The success of metal‐based anticancer therapeutics in the treatment of cancer is best exemplified by cisplatin. Currently used in 32/78 cancer regimens, metal‐based drugs have a clear role in cancer therapy. Despite this, metal‐based anticancer therapeutics are not without drawbacks, with issues such as toxic side effects and the development of resistance mechanisms. This has led to investigations of other metal‐based drug candidates such as auranofin, a gold‐based drug candidate as well as ruthenium‐based candidates, NAMI‐A, NKP‐1339 and TLD‐1433. All are currently undergoing clinical trials. Another class of complexes under study are rhenium‐based; such complexes have undergone extensive in vitro testing but only nine have been reported to display antitumour in vivo activity, which is a necessary step before entering clinical trials. This review will document, chronologically, the rhenium‐based drug candidates that have undergone in vivo testing and the outlook for such complexes.

## Graphical abstract

**Beyond cisplatin**: The use of animal models, otherwise known as in vivo testing, is a crucial step in drug development. To date there have only been nine reported cases of rhenium‐based complexes undergoing in vivo testing. This concept article explores the history of these complexes.

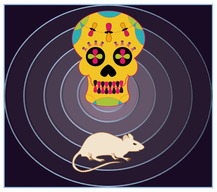

## Introduction

The search for new and more effective anticancer drugs is an area of high importance. Current metal‐based treatments, such as cisplatin, have limitations associated with them in the form of unwanted side effects and chemotherapeutic resistance.^1^ In recent years, research dedicated to the development of new pharmaceuticals has investigated a range of different complexes that contain metal centre atoms, such as Ag, Au, Ir, Pd, Os, Ru, Rh, Re and Ti.^2^, ^3^

In particular, Re‐based complexes have recently drawn interest. Typically, Re has been associated with nuclear imaging applications due to the medical relevance of the isotope ^99m^Tc, which is used in approximately 90 % of all nuclear imaging applications.^4^ In 1998, Alberto et al. first reported the synthesis of [^99m^Tc(H_2_O)_3_(CO)_3_]^+^ which gave rise to the development of ligand systems coordinated to the [M(CO)_3_]^+^ core.^5^ This research included an investigation of Re^I^ tricarbonyl analogues as well as Tc.

The in vitro antiproliferative effects of rhenium‐based complexes have been extensively detailed in previous reviews.^6^, ^7^, ^8^ This also includes the photophysical and photobiological properties and their applications in photodynamic therapy (PDT) and photoactivated chemotherapy (PACT). Despite this, there have only been a total of nine rhenium‐based complexes that have been reported to display antitumour in vivo activity.^9^, ^10^, ^11^, ^12^, ^13^, ^14^, ^15^, ^16^ On the path to the development of a clinically used drug, the use of animal models is a crucial step before human trials can begin. This review will solely discuss the timeline of rhenium‐based complexes that have advanced to the in vivo stage of testing and the future of such complexes.

## Re^III^ Cluster

**Figure 1:**
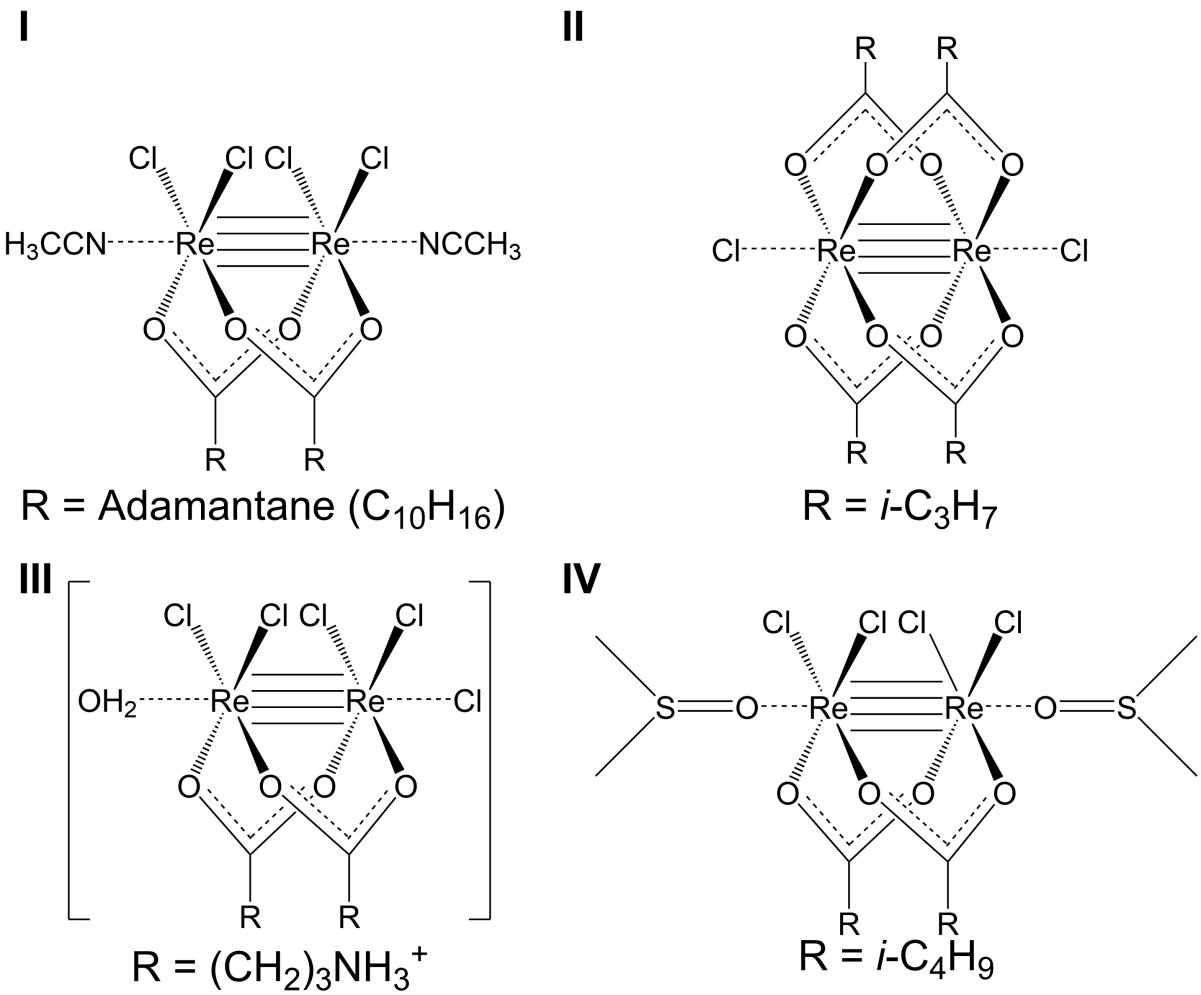
**I**) *cis*‐Rhenium(III) adamantane chloride; **II**) dichloro‐μ‐isobutyratodirhenium(III); **III**) *cis*‐[Re_2_(GABA)_2_Cl_4_]Cl^.^H_2_O {GABA=γ‐aminobutyrate); **IV**) *cis*‐tetrachloro‐dipivalato dirhenium(III).

In 1983, the first example of a Re^III^ cluster complex, Re_2_(EtCOO)_2_Br_4_(H_2_O)_2_, being used to treat tumour‐bearing mice was reported by Eastland et al.^17^ Against sarcoma S‐180, leukaemia P‐388 and melanoma B‐16, the complex showed little antitumour activity with the exception being B‐16 which displayed some activity at very high doses. The complex itself displayed poor stability and decomposed readily in aqueous solution to insoluble rhenium oxides; antitumour effects were hence only observed when large doses were administered. Later efforts into developing rhenium‐based anticancer chemotherapeutics also focused on Re^III^ clusters with a quadruple bond. In total, there are four complexes of this type (Figure 1) that have undergone successful antitumour in vivo testing.^10^, ^12^, ^14^, ^16^ In all cases, the animal models used were Wistar rats inoculated with tumour carcinoma Guerink (T8) cells. Each complex was administered in liposomal form to address the poor aqueous solubility and low stability of the compounds.

The earliest example of rhenium‐based complexes displaying antitumour in vivo activity was complex **I** (Figure 1) which was reported in 2006 by Shetmenko et al.^16^ The co‐administration of **I** and cisplatin produced a synergistic effect leading to an enhanced cisplatin action on tumour growth. The succeeding study showed a similar synergistic effect between **II** and cisplatin.^14^ In previous in vivo experiments, **II** had been shown to be a unique stabilizer of red blood cells against acid haemolysis and it revealed antianaemic effects.^14^, ^18^

In the case of **III** (Figure 1), the complex displayed stronger antitumour effects than **I** and **II** by 28–30 % and 20–25 %, respectively, when administered on its own.^12^ It was proposed that due to the cationic nature of **III** it might be able to interact and bind with DNA more efficiently via electrostatic attraction, which is also well documented for platinum‐based complexes and the formation of 1,2‐intrastrand adducts.^19^ When co‐administered with cisplatin, a synergistic effect was observed but this was weaker than that of **I** and **II**. Complex **IV** (Figure 1) was the most recent complex with a Re^III^ quadruple bond to have displayed antitumour in vivo activity.^10^ Building on the previous finding that the cationic species **III** covalently binds with DNA model bases, a number of studies were conducted to investigate how **IV** interacted with DNA. Using electrophoresis mobility shift assays, viscosity measurements, melting temperature experiments and electronic absorption titrations it was postulated that **IV** can form covalent intrastrand crosslinks and kinks and is able to unwind supercoiled DNA.^10^ Additionally, electrophoresis experiments showed that the redox activation of DNA when cleaved by **IV** might relate to the anticancer activity of the latter. During in vivo testing, the antitumour activity when administered without any additional chemotherapeutic agents surpassed that of **I**, **II** and **III**. When co‐administered with cisplatin, there was remarkable tumour suppression and, in some cases, complete elimination of the tumour. When considering previous structures, the increasing hydrophobicity of the bridging ligands in **IV** appeared to be a successful approach for increasing the antitumour activity of Re^III^ clusters.

To date, there have been no further examples of Re^III^ complexes displaying in vivo antitumour activity but the previous research has demonstrated successful synergistic effects when administered in combination with cisplatin. Additionally, rhenium‐platinum synergism has been reviewed giving insights into the future outlook of these systems.^20^

## Re^I^ Tricarbonyl

In more recent years, the focus has shifted to rhenium(I) tricarbonyl complexes. This reflects that such compounds have the anticancer properties of a chemotherapeutic drug and additionally feature suitable photophysical properties, which render them ideal for treatments such as photodynamic therapy (PDT) as well as photoactivated chemotherapy (PACT).^7^, ^8^ Furthermore, such complexes possess luminescent properties making them observable in the cellular environment through techniques such as confocal fluorescence microscopy. To date, there have only been a small number of reported cases of Re^I^ tricarbonyl complexes displaying in vivo anticancer activity.^9^, ^11^, ^13^, ^15^

Collery et al. first reported antitumour in vivo testing of a Re^I^ tricarbonyl complex in 2015.^11^ This involved a Re atom coordinated equatorially to two selenium atoms with the complex being stable, water soluble and lipophilic. The cytotoxic and antitumour effects of selenium have been well documented previously.^21^, ^22^, ^23^, ^24^, ^25^ Complex **V** had previously shown impressive cytotoxicity against the MCF‐7 breast cancer cell line with an IC_50_ value of 4.75 μM.^25^ In a more recent study, the IC_50_ value against the MCF‐7 breast cancer cell line was reported to be 51.4±3.0 μM (Table 1).^24^ Additionally, using flow cytometry, complex **V** (10 μM) was observed to slow down cell proliferation of the MDA‐MB‐231 breast cancer cell line and it induced continued antiproliferative effects even after the complex was removed. Potential targets of **V** were investigated with an observed decrease in the production of IGF‐1, VEGF‐A and TGF‐β1 in MDA‐MB‐231 cells when treated with **V**.^24^ The downregulation of biomarkers was thought to cause a selective inhibitory effect in cancer cells. When exposed at high concentrations (400 μM) to MCF‐7s (sensitive), MCF‐7R (resistant) and MCF‐7 MDR (multidrug‐resistant) breast cancer cell lines, the potential targets of **V** were measured using inductively coupled plasma mass spectrometry (ICP‐MS).^26^ These analyses revealed an efflux of Re out of the nucleus, indicating that it was necessary to maintain a continuous dose of **V** to malignant cells, which could be achieved via oral administration. It was determined that a dose of 10 mg/kg of **V** was safe for mice that were treated daily over the course of 4 weeks either orally or intraperitoneally.^11^, ^27^ At this concentration, **V** was shown to reduce tumour growth in mice transplanted with MDA‐MB231 Luc+ human breast tumour cells. To date, **V** remains the most extensively studied Re^I^ tricarbonyl complex, with numerous in vitro and in vivo studies performed.

**Table 1:** IC_50_ [μM] values of Re^I^ tricarbonyl complexes.^8^, ^9^, ^13^, ^28^

	Complex				
Cell line	**V** ^[a]^	**VI** ^[b]^	**VII** ^[b]^	**VIII** ^[b]^	**IX** ^[a]^
KB‐3‐1	–	–	–	–	0.92±0.2
KBCP20	–	–	–	–	1.6±0.4
A2780	–	–	–	–	2.2±0.2
A2780CP70	–	–	–	–	3.0±0.7
A549	133.2±4.3	2.2±0.2	1.1±0.1	0.8±0.1	6.7±4.9
AF49CisR	–	2.1±0.1	8.3±0.1	2.1±0.1	5.4±1.8
H460	–	–	–	–	4.5±0.7
H460CisR	–	–	–	–	5.3±2.9
MRC‐5	–	–	–	–	4.1±0.9
HeLa	126.4±2.8	1.8±0.2	1.6±0.1	1.8±0.1	1.2±0.2
U2SO	–	–	1.4±0.1	1.1±0.1	–
MCF‐7	51.4±3.0	2.2±0.3	11.8±1.3	1.5±0.1	–
PC3	59.4±3.8	–	>25	1.1±0.1	–
HepG2	–	–	>25	1.6±0.1	–
LO2	–	–	7.5±0.1	2.0±0.1	–
HLF	–	12.7±0.8	–	–	–
MDA‐MB‐231	48.5±2.8	–	–	–	–
HT‐29	47.5±0.9	–	–	–	–

[a] 72 h incubation/MTT assay [b] 44 h incubation/MTT assay.

**Figure 2:**
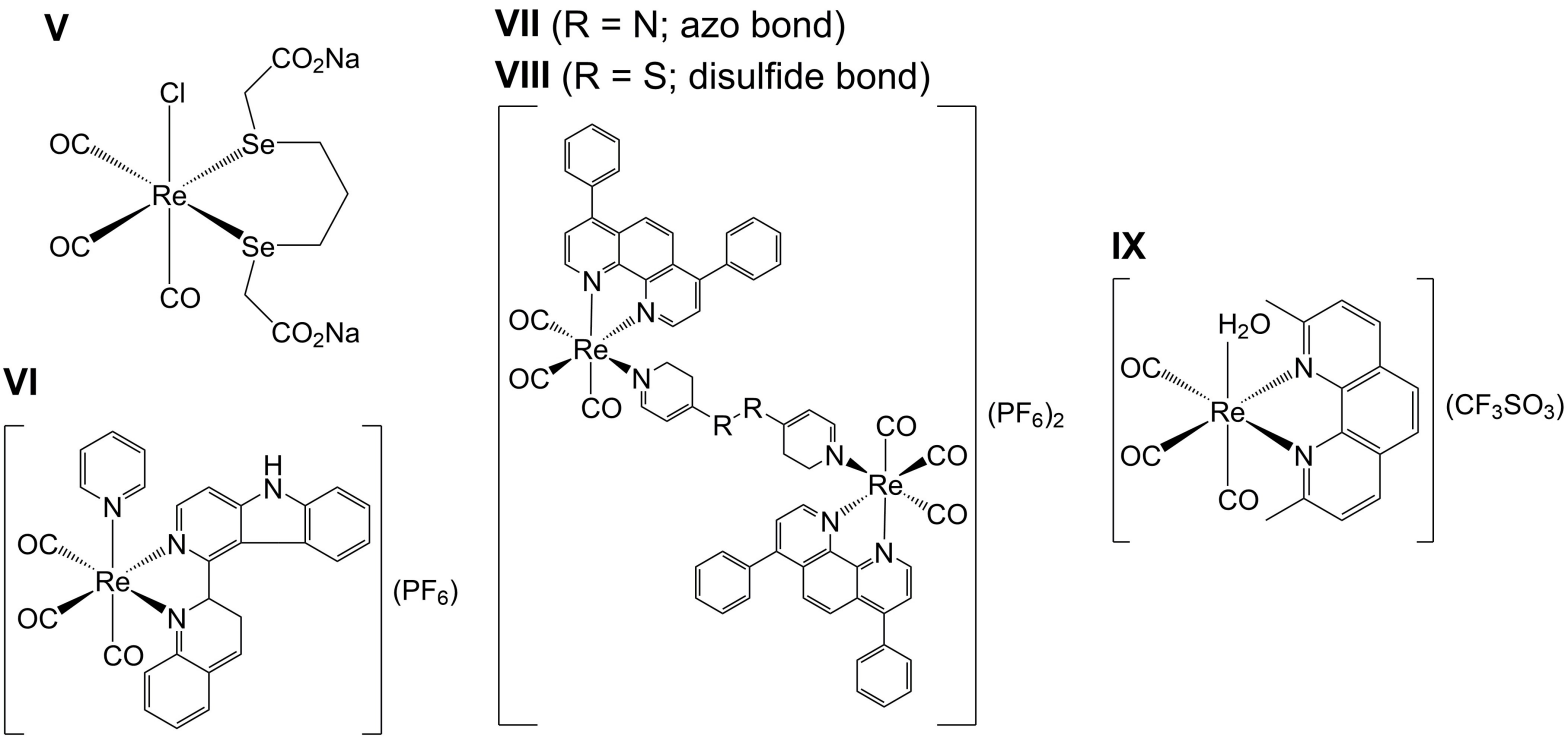
**V**) *fac*‐[Re(CO)_3_(2,2’‐(propane‐1,3‐diylbis(selanediyl))diacetate)] (disodium salt); **VI**) *fac*‐[Re(CO)_3_(1‐quinolin‐2‐yl)‐*9H*‐pyrido[3,4‐*b*]indole)(pyridine)](PF_6_); **VII**) *fac*‐[Re_2_(CO)_6_(4,7‐diphenyl‐1,10‐phenanthroline)_2_ 4,4′‐azopyridine](PF_6_)_2_; **VIII**) *fac*‐[Re_2_(CO)_6_(4,7‐diphenyl‐1,10‐phenanthroline)_2_ 4,4′‐dithiodipyridine](PF_6_)_2_; **IX**) *fac*‐[Re(CO)_3_(2,9‐dimethyl‐1,10‐phenanthroline)(H_2_O)](CF_3_SO_3_).

In 2019, He et al. investigated two Re complexes that bear pyridine and β‐carboline ligands.^13^ Measurements of photophysical properties indicated that phosphorescence was pH‐dependent whilst confocal laser scanning microscopy (CLSM) demonstrated preferential uptake into the lysosomes. The stability of the complex in phosphate‐buffered solution (PBS)/DMSO (4 : 1, *v/v*) was measured by UV‐Vis spectroscopy over the course of 24 h, and this showed minimal changes, indicating good stability. The in vitro anticancer activity was assessed against a range of cell lines (A549R, Hela, MCF‐7, HLF, Table 1). Both complexes showed activity against the investigated cell lines, with the best results achieved against cisplatin‐resistant human lung carcinoma cells (A549R). Complex **VI** (Figure 2) displayed higher activity with an IC_50_ value of 2.1±0.1 μM (Table 1), which meant it was selected for further in vivo testing. Using nude mice bearing A549 tumour xenografts, the activity of **VI** (5 mg kg^−1^) was tested over the course of 21 days. When compared with the control group, a 60 % reduction in average tumour volume was recorded. Mechanistic studies revealed that **VI** caused lysosomal dysfunction, which in turn impaired lysosomal enzymatic activity. Additionally, **VI** was reported to activate autophagy but due to the impaired lysosomal hydrolases this process cannot be carried out, which leads to accumulation of impaired organelles, proteins and biomacromolecules in the lysosomes and this ultimately induces cell death via caspase‐independent apoptosis. This is the first reported Re^I^ complex that induces cell death via autophagy.

Following this study, the research group of Mao and Tan, reported on the anticancer in vivo activity of two binuclear Re^I^ tricarbonyl complexes (Figure 2).^9^ The reduction of the azo bond and breakage of the disulfide bond using GSH was confirmed and measured using electrospray ionization mass spectrometry (ESI‐MS), ^1^H NMR, absorption and emission spectroscopy as well as emission lifetime. Similar complexes were reported to be stable for at least 48 h in PBS when measured with UV‐Vis spectroscopy and ESI‐MS.^29^ Both **VII** and **VIII** were dissolved in DMSO before experiments indicating the complexes had poor water solubility. The in vitro anticancer activity was assessed against a range of cancerous and normal cells lines (Table 1). Both displayed increased anticancer activity against the range of cell lines, with IC_50_ values surpassing that of cisplatin and **VIII** being the more potent of the two. In addition, the complexes displayed high anticancer activity against the A549R cells line with IC_50_ values of 8.3±0.1 and 2.1±0.1 for complexes **VII** and **VIII** respectively (Table 1). This indicates that they may be able to overcome cisplatin induced resistance. Accumulation of both **VII** and **VIII** was shown to be in the mitochondria of HeLa cells which was determined using CLSM and further confirmed using ICP‐MS. Additionally, the complexes were shown to cause oxidative stress and mitochondrial dysfunction with a slow‐down of mitochondrial bioenergetics. Furthermore, they were reported to affect GSH metabolism and redox homeostasis via impacted redox‐related enzymes and species. Both complexes were also shown to induce apoptosis and necroptosis. Using HeLa xenografts that were established by subcutaneous injections into BALB/c nude mice, the anticancer in vivo activity was assessed. Two intratumoural injections of either **VII** or **VIII** (5 mg/kg) in PET diluent (6 % polyethylene glycol 400, 3 % ethanol, and 1 % Tween 80 in phosphate buffered saline, PBS) were performed at days 0 and 9 over the course of the 19‐day experiment. The complexes **VII** and **VIII** were both able to inhibit tumour growth but did not surpass the activity of cisplatin. By using body weight, survival rate and the staining of organs with hematoxylin and eosin (H & E), the side effects of both complexes were assessed. No obvious pathological changes were observed between the organs from each group, which indicates that both **VII** and **VIII** do not induce undesirable side effects.

Most recently, Wilson et al. tested a library of complexes bearing the general structure *fac*‐[Re(CO)_3_(diimine)(H_2_O)](CF_3_SO_3_), whereby the aqua ligand helps to improve water solubility.^28^ In this study, complex **IX** (Figure 2) revealed promising in vitro anticancer activity against a range of cancer cell lines and was therefore progressed to the in vivo testing stage (Table 1).^15^ Analysis of blood and urine metabolites from C57B16 mice using high‐performance liquid chromatography inductively coupled plasma mass spectrometry (HPLC‐ICP‐MS) displayed the intact aqua and chloride forms of **IX**, indicating sufficient in vivo stability. Intracellular uptake was identified through the fractionation of cells and analysis of the separated components using ICP‐MS. The results demonstrate that the complex was taken up by both wild‐type (A2780) and cisplatin‐resistant (A2780CP70) ovarian cancer cell lines. Previous in vitro anticancer studies showed that the IC_50_ value of **IX** against the A2780 and A2780CP70 cell lines is 2.2±0.2 and 3.0±0.7 μM, respectively (Table 1). Interestingly, the intracellular localization identified **IX** in the mitochondria whereas a previous study with confocal fluorescence microscopy concluded that **IX** induced cytoplasmic vacuolization.^14^ These contradictory results reflect that the emissive properties of **IX** were altered and/or quenched in the different biological environments through either variable pH or interaction with biologically endogenous ligands. These results indicate that localization data that are obtained for complexes using confocal fluorescence microscopy can be unreliable and should be confirmed with other techniques, such as ICP‐MS. This phenomenon was previously observed by Alberto et al. who reported fluorescence quenching of Re‐based conjugates by DNA intercalation.^30^, ^31^ In both cases Re uptake was confirmed by ICP‐MS. Further in vivo testing of **IX** was performed using ovarian cancer patient‐derived xenografts (PDX) implanted subcutaneously in the right flank of NOD‐scid gamma (NSG) mice. A range of doses were used (10, 20 and 40 mg/kg) with no difference observed in tumour growth between the treatments, which suggests that the maximum biological effect was already achieved with a dose of 10 mg/kg. Furthermore, only small differences in organ weights were observed for the mice indicating that **IX** did not cause any adverse side effects. In addition, microscopy following staining with H & E revealed no apparent tissue damage between treated and control mice. This most recent identification of a rhenium‐based complex with favourable antitumour effects suggests that future studies of rhenium‐based complexes may reveal further promising drug candidates.

## Conclusions

Initial tests of rhenium‐based complexes as anticancer drugs focused on Re^III^ clusters, which displayed synergistic effects when co‐administered with cisplatin. However, despite initially promising results, such Re^III^ clusters have yet to advance into human clinical trials. In more recent years, the focus of research has shifted towards Re^I^ tricarbonyl complexes. The examples discussed in this review present variable intracellular localization and appear to have distinct mechanisms of action. As such, they are interesting candidates for further testing.

Rhenium‐based complexes face a similar set of challenges as other metal‐based drug candidates, with a focus on maximizing the effect of the primary mechanism of action whilst diminishing secondary and/or off target mechanisms. The classification of metal‐based drugs by the primary mechanisms was recently reviewed and discussed in depth.^32^ Additionally, full characterization of such complexes at relevant conditions prior to further biological testing is also of worthwhile, to better constrain and understand the biologically active species. The importance of this approach was recently shown in a study of two ruthenium‐based complexes.^33^ For rhenium‐based anticancer drug candidates, such challenges can be addressed by the acquisition of more data on the physiological effects of the complexes, as a first step towards eventual clinical trials. The solution to this is one of time, effort and resources and based on the increasing number of recent reports on rhenium‐based drug candidates this appears to be happening. In this context, the development of ruthenium‐based anticancer agents can be taken as inspiration, as the their development first began in the 1980s and they are currently in clinical trials.^34^, ^35^ Whilst non‐radioactive rhenium‐based complexes are currently not undergoing clinical trials, this progression is likely to be only a matter of time, given the significant ongoing efforts that are dedicated toward exploring Re^I^ tricarbonyl complexes and their range of interesting chemotherapeutic and photophysical properties.

## Conflict of interest

The authors declare no conflict of interest.
